# Clinical outcomes of different 17β-estradiol drug regimens and their impact on endometrial receptivity

**DOI:** 10.3389/fendo.2025.1639481

**Published:** 2025-09-19

**Authors:** Beining Luo, Xuehong Zhu, Ni Tang, Zhong Lin, Jun Yao, Zhengqin Chen, Zhuo Liang, JinXiang Wu, Bingsheng Huang, Pinxiu Huang

**Affiliations:** ^1^ Center of Reproductive Medicine, The First Affiliated Hospital of Guilin Medical University, Guiling, China; ^2^ Department of Reproductive Medicine, Guangxi Zhuang Autonomous Region Reproductive Hospital, Nanning, China; ^3^ Center of Reproductive Medicine, Guangzhou Women and Children’s Medical Center-Liuzhou Hospital, Liuzhou, Guangxi, China; ^4^ Department of Reproductive Medicine, the Second Affiliated Hospital of Fujian Medical University, Quanzhou, Fujian, China; ^5^ Affiliated Hospital of Youjiang Medical University for Nationalities, Baise, Guangxi, China; ^6^ Key Laboratory of Clinical Diagnosis and Treatment Research of High Incidence disease in Guangxi, Baise, Guangxi, China

**Keywords:** Femoston, Progynova, HRT, frozen-thawed embryo, endometrial receptivity endometrial receptivity

## Abstract

**Background:**

Clinically, it has been observed that vaginal administration of the same dose of Micronized 17-beta estradiol hemihydrate (the Estradiol tablets of Femoston) significantly increases serum estradiol levels compared to oral administration. However, the clinical outcomes associated with this route of administration remain unclear. Additionally, the concentration of estradiol in endometrial tissue following vaginal administration of Micronized 17-beta estradiol hemihydrate (M17EH), as well as its potential impact on endometrial receptivity, has been poorly investigated.

**Objective:**

To explore the relationship between different drug regimens of M17EH hormone replacement (HRT) and serum estradiol level, endometrial thickness and embryo implantation outcome in patients with thin endometrium during the frozen-thawed embryo transfer (FET) cycle, and to analyze the estradiol concentration in endometrial tissue of oral and vaginal administration of M17EH and its influence on endometrial receptivity.

**Method:**

A retrospective analysis was conducted on patients with thin endometrium. Subjects were divided into three groups based on different estrogen formulations and administration routes: Group A [oral Estradiol Valerate Tablets (Progynova)], Group B (oral M17EH), and Group C (oral combined with vaginal M17EH). Endometrial thickness, and clinical outcomes were compared across the three groups. For Groups B and C, endometrial tissue samples were collected five days after progesterone conversion. Estradiol concentration in tissues was detected and the endometrial receptivity markers [leukemia inhibitory factor, (LIF) and Mucins, (Muc1)] were evaluated.

**Results:**

Serum estradiol levels and endometrial thickness in Group C were significantly higher than those in the other two groups (P < 0.05). While there were no statistically significant differences in abortion rate, and live birth rate among the three groups, the live birth rate was highest in Group C. Estradiol concentration in the endometrium was significantly higher following vaginal administration of M17EH compared to oral administration (P<0.05). No significant differences were observed in the expression of endometrial receptivity markers (LIF and MUC1) between oral and vaginal administration groups.

**Conclusion:**

In FET cycles, a HRT regimen combining oral and vaginal administration of Micronized 17-beta estradiol hemihydrate is more conducive to endometrial growth. Although vaginal administration results in higher estrogen levels, it does not appear to compromise endometrial receptivity.

## 1 Introduction

In frozen embryo transfer (FET) cycles, the clinical pregnancy rate, ongoing pregnancy rate, and live birth rate are comparable between natural cycle and hormone replacement therapy (HRT)-based endometrial preparation methods (1, 2). HRT is widely used for endometrial preparation in FET due to its advantages of reducing hospital visit frequency, low cycle cancellation rate, convenient monitoring, and flexible scheduling for patients, which aligns well with clinical practice and patient needs. One of the critical prerequisites for successful frozen embryo transfer (FET) is adequate endometrial thickness, as thin endometrium represents a major contributor to embryo implantation failure. The definition of thin endometrium is generally established as endometrial thickness <8 mm on the human chorionic gonadotropin (HCG) administration day in *in vitro* fertilization-embryo transfer (IVF-ET) cycles, and <7 mm on the progesterone conversion day in thawing cycles (3).

The primary etiology of thin endometrium is insufficient endometrial proliferation, which leads to failure in achieving ideal endometrial thickness. Endometrial thickness is widely regarded as a surrogate marker for endometrial functional status and a predictor of endometrial receptivity. The thickness of the endometrium is closely associated with pregnancy outcomes. Multiple studies, including our previous research, have demonstrated that the pregnancy rate increases proportionally with endometrial thickness, exhibiting a linear positive correlation (4, 5).

Currently, a key challenge in reproductive medicine lies in enhancing endometrial receptivity for patients with thin endometria. For individuals with endometrial thinning—particularly those undergoing natural cycles or whose hormone replacement therapy (HRT) with Estradiol Valerate Tablets (Progynova) failed to achieve optimal thickness, leading to cycle cancellation—improving endometrial thickness remains a critical clinical dilemma.

In recent years, Femoston (containing Micronized 17-beta estradiol hemihydrate/Micronized 17-beta estradiol hemihydrate compound dydrogesterone) has been incorporated into hormone replacement therapy (HRT) cycles (6). Notably, its vaginal administration bypasses gastrointestinal digestion and degradation of oral medications, allowing direct absorption through vaginal mucosa into the bloodstream. This approach has offered new hope to patients with endometrial hypoplasia. Research reports and clinical observations indicate that vaginal administration of Micronized 17-beta estradiol hemihydrate results in significantly higher serum estradiol levels than oral administration. However, the impact of this method on endometrial receptivity remains unclear.

This study retrospectively analyzed the association between hormone replacement therapy (HRT) with Estradiol Valerate Tablets (Progynova) and different administration regimens of Micronized 17-beta estradiol hemihydrate (Femoston) on endometrial thickness and clinical outcomes. It also explored the effects of varying Micronized 17-beta estradiol hemihydrate regimens on estradiol levels in endometrial tissue and molecular markers of endometrial receptivity, aiming to provide clinical guidance for medication strategies.

## 2 Materials and methods

### 2.1 Research object

This study selected the frozen-thawed embryo transfer (FET) cycles with hormone replacement therapy (HRT) regimen from January 1, 2016 to December 31, 2023 at the First Affiliated Hospital of Guilin Medical University. Inclusion criteria: 20–40 years old; previous natural cycles showed endometrium failed to reach 7 mm leading to FET cycle cancellation; at least 2 frozen-thawed embryos available including at least one high-quality embryo. Exclusion criteria: coronary heart disease, hypertension, thrombophilia; hypersensitivity to estrogen medications; hydrosalpinx, intrauterine fluid accumulation, endometritis, endometrial polyps, endometrial hyperplastic lesions. The study protocol received ethical approval from the Institutional Review Board of Guilin Medical University Affiliated Hospital. High-quality embryos were defined as Istanbul consensus (7). The high quality blastomere embryos: normal cell number, fragments ≤20%, and slightly uneven blastomeres. The high quality blastocyst: expanded blastocyst (blastocyst cavity occupies the whole embryo), inner cell mass (many and compact cells) and trophoblast cells (continuous cell layer and clear structure).

### 2.2 Research method

The subjects received the following medication regimens starting from the 2nd to 3rd day of menstruation. Group A: Oral administration of Estradiol Valerate Tablets (Progynova, Bayer) at 4 mg/d for 7 days, followed by 6 mg/d for the subsequent 7 days. Group B: Oral administration of Micronized 17-beta estradiol hemihydrate (Femoston, Estradiol tablets, 2/10mg, Solvay pharmaceuticals B.V.) at 4 mg/d for 7 days, then 6 mg/d for 7 days. Group C: Oral Micronized 17-beta estradiol hemihydrate at 4 mg/d for 7 days, followed by vaginal administration at 2 mg/d for 7 days. After 14 days, when endometrial thickness reached ≥8 mm, serum E_2_ and P were measured. If P was <1.0ng/mL, treatment included 10 mg Dydrogesterone (Duphaston, Abbott Biologicals) orally three times daily, 0.2g progesterone soft capsules (Utrogestan,Cyndea Pharma,S.L) vaginally twice daily, with estrogen continued as before. Embryo transfer was performed 3–5 days after endometrial preparation, with 1–2 embryos (including at least 1 high-quality embryo) transferred.

### 2.3 Collection in tissue of endometrial implantation window

We collected the above cases where FET was canceled due to personal factors. Five days after transformation, endometrial tissues were obtained from 5 cases in Group B and 5 cases in Group C. Each case had three specimens preserved: one fixed with formaldehyde for immunohistochemistry, another stored at -80°C for mRNA extraction, and the third washed with PBS to remove surface blood. The tissue samples were mixed with appropriate PBS, homogenized, centrifuged, and the supernatant was collected for subsequent use.

### 2.4 Detection of endometrial receptivity molecular markers

Immunohistochemistry was performed following standard immunohistochemical procedure. Muc1 antibody (Abcam, ab109185, 1:1000 dilution) and LIF antibody (Proteintech, 26757-1-AP, 1:1000 dilution) were used to detect protein expression. Total mRNA was extracted and reverse-transcribed using a Takara kit, and qPCR was performed to analyze Muc1and LIF expression according to the kit instructions.

### 2.5 Determination of E2 level in endometrial tissue by ELISA kit instruction

Endometrial E_2_ concentrations were measured by radioimmunoassay (RIA) after tissue homogenization, using a method adapted from Tourgeman (7). The tissue E_2_ RIA protocol was as follows: A tritiated E_2_ internal standard (1000 disintegrations per minute) was added to the homogenate, followed by steroid extraction with hexane/ethyl acetate. The organic solvent was evaporated under a nitrogen stream at 37°C, and the residue was redissolved in assay buffer. Duplicate aliquots were subjected to RIA, while a third aliquot was counted to assess procedural losses. The RIA procedure for E_2_ measurement followed the same protocol as serum E_2_ analysis described previously.

### 2.6 Observation and analysis index

General clinical data included age, BMI, composition ratio of infertility causes, endometrial thickness on the day of endometrial transformation, serum E_2_, endometrial tissue E_2_ level, and the expression of endometrial receptivity molecules LIF and Muc1. The clinical pregnancy is confirmed by B-ultrasound that there is a gestational sac in the uterine cavity after embryo transfer 25 days. Pregnancy loss before 12 weeks was defined as abortion. The clinical pregnancy rate was calculated as (number of clinical pregnancy cycles/total transplantation cycles) × 100%; the abortion rate as (number of abortion cycles/number of clinical pregnancy cycles) × 100%; and the live birth rate as (number of live birth cycles/total number of transplantation cycles) × 100%.

### 2.7 Statistical analysis

Statistical analysis was conducted using SPSS 13.0 software. Appropriate statistical methods included Student’s t-test, chi-square test, Fisher’s exact test, and analysis of variance (ANOVA). Statistical significance was set at *P* < 0.05.

## 3 Result

**Figure 1:**
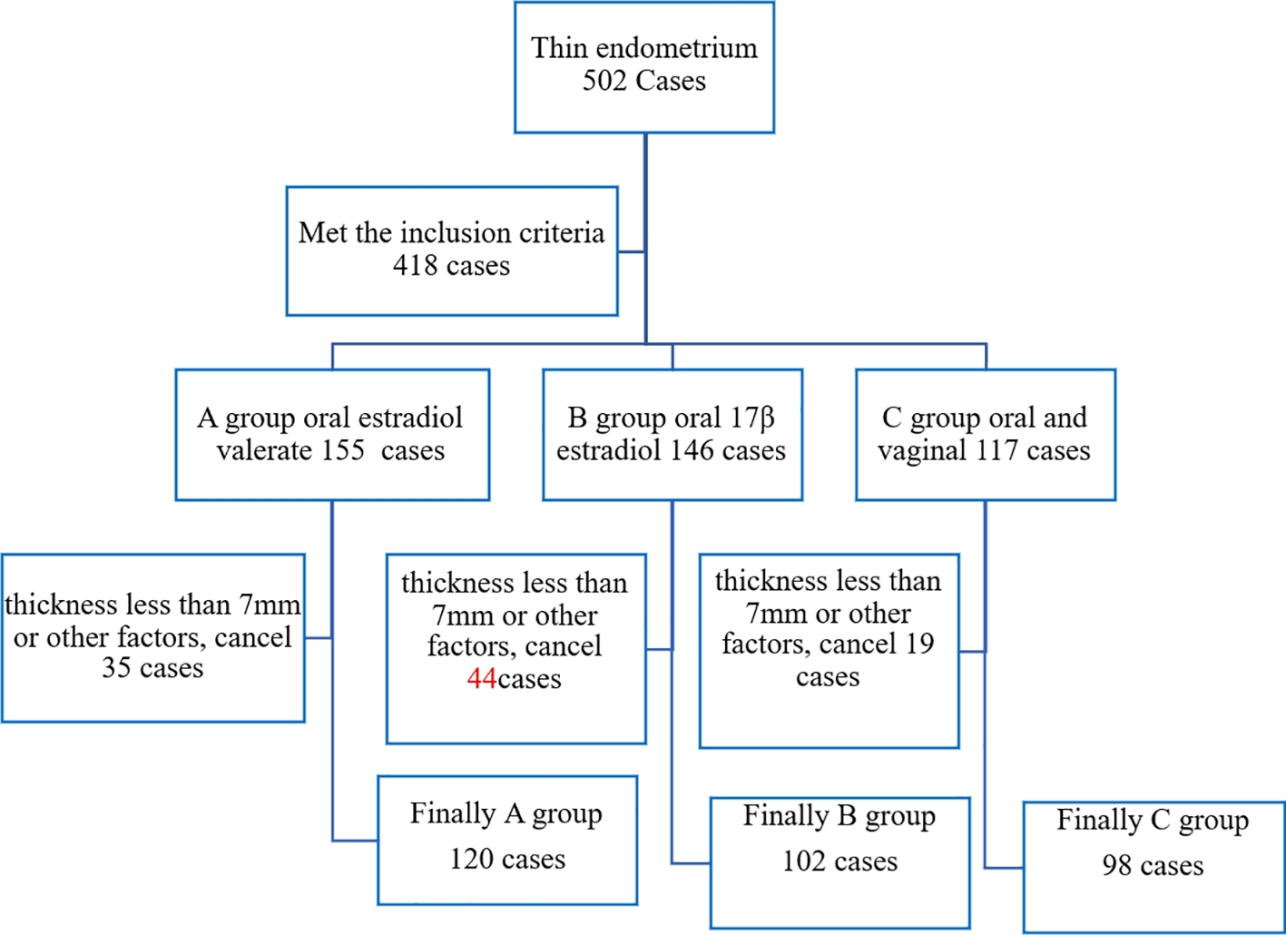
Flow chart of case screening.

A total of 502 cases underwent hormone replacement therapy (HRT) due to thin endometrium (natural cycle thickness <7 mm). According to different HRT regimens, 418 cases were divided into Group A [oral Estradiol Valerate Tablets (Progynova)], Group B (oral Micronized 17-beta estradiol hemihydrate), and Group C (oral combined with vaginal Micronized 17-beta estradiol hemihydrate). Cycles were canceled due to endometrial thickness <7 mm or other factors, leaving 320 cases. Finally, there were 120 cases in Group A, 102 cases in Group B, and 98 cases in Group C, as illustrated in **Figure 1**.

There were no statistically significant differences in age, BMI, basal FSH, and infertility duration among the three groups (analysis of variance), as presented in **Table 1**.

**Table 1:** General clinical data of three groups of patients.

Group	A group (n=120)	B group (n=102)	C group (n=98)	*P*
Age (years)	33.02 ± 4.50	32.76 ± 5.20	32.89 ± 4.65	>0.05
Infertility age	4.23 ± 3.12	4.56 ± 3.49	5.08 ± 3.98	>0.05
Basic FSH	6.54 ± 3.56	6.75 ± 4.52	6.79 ± 521	>0.05
BMI	21.56 ± 2.65	22.04 ± 2.15	21.96 ± 2.89	>0.05

On the day of endometrial transformation, the endometrial thicknesses of Groups A, B, and C were 8.12 ± 1.49 mm, 8.56 ± 1.68 mm, and 9.56 ± 1.68 mm, respectively. The endometrial thickness in Group C was significantly higher than that in Groups A and B (ANOVA, P<0.05). There were no statistical differences in the number of transferred embryos, the proportion of D3 embryos, and D5 blastocysts transferred among the three groups. The live birth rates of Groups A, B, and C were 40.83% (49/120), 45.10% (46/102), and 52.04% (51/98), respectively. No statistical differences were observed in the clinical pregnancy rate, abortion rate, and live birth rate among the three groups, as shown in **Table 2**.

**Table 2:** Clinical outcomes of three groups of patients.

Group	A group (n=120)	B group (n=102)	C group (n=98)	*P*
Endometrial thickness(mm)	8.12 ± 1.49	8.56 ± 1.68	9.56 ± 1.68	<0.05
ET embryo number	1.56 ± 0.65	1.52 ± 0.56	1.55 ± 0.35	>0.05
ET D3 embryo ratio	55.00(66/120)	56.88(57/102)	57.14(56/98)	>0.05
ETD5 Blastocyst ratio	45.00(54/120)	44.12(45/102)	42.86(42/98)	>0.05
Clinical Pregnancy rate	47.50(57/120)	51.96(53/102)	60.20(59/98)	>0.05
Abortion rate	14.03(8/57)	13.20(7/53)	13.56(8/59)	>0.05
Live birth rate	40.83(49/120)	45.10(46/102)	52.04(51/98)	>0.05

**Figure 2:**
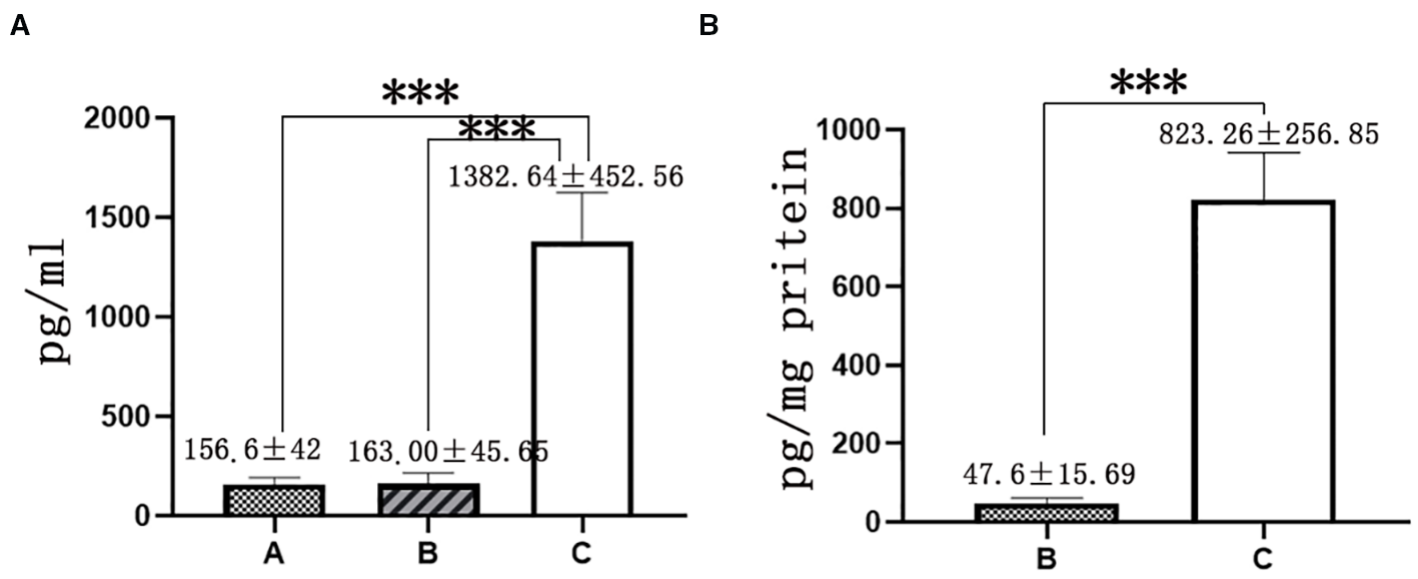
E2 level in serum and endometrial tissue. **(A)**: E2 level in serum, **(B)** E2 level in endometrial tissue, **(A)** group oral Estradiol Valerate Tablets; **(B)** oral Micronized 17-beta estradiol hemihydrate; **(C)** group oral and vaginal; *** represents P<0.001.

The serum E_2_ levels in the three groups were 156.6 ± 42.25, 163.00 ± 45.65, and 1382.64 ± 452.56 pg/ml, respectively (ANOVA, P < 0.05). Group C showed significantly higher serum E_2_ levels than Groups A and B (P < 0.05), while no statistical difference was observed between Groups A and B. The endometrial tissue E_2_ levels in Groups B and C were 47.60 ± 15.69 and 823.26 ± 256.85 pg/mg protein, respectively. Group C exhibited significantly higher endometrial tissue E_2_ levels than Group B (P < 0.05), as illustrated in **Figure 2**.

**Figure 3:**
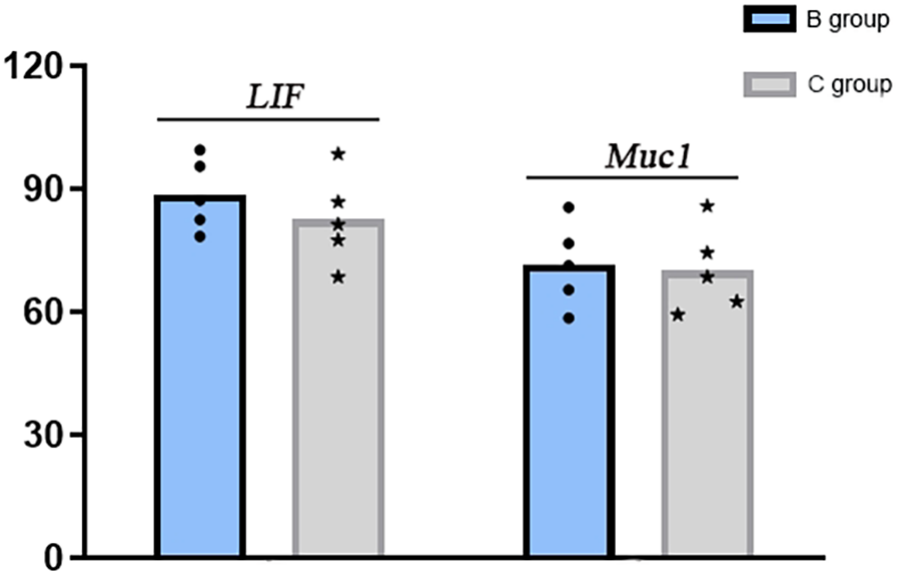
mRNA levels of endometrial receptivity markers LIF and Muc1. **(B)** oral Micronized 17-beta estradiol hemihydrate; **(C)** group oral and vaginal.

**Figure 4:**
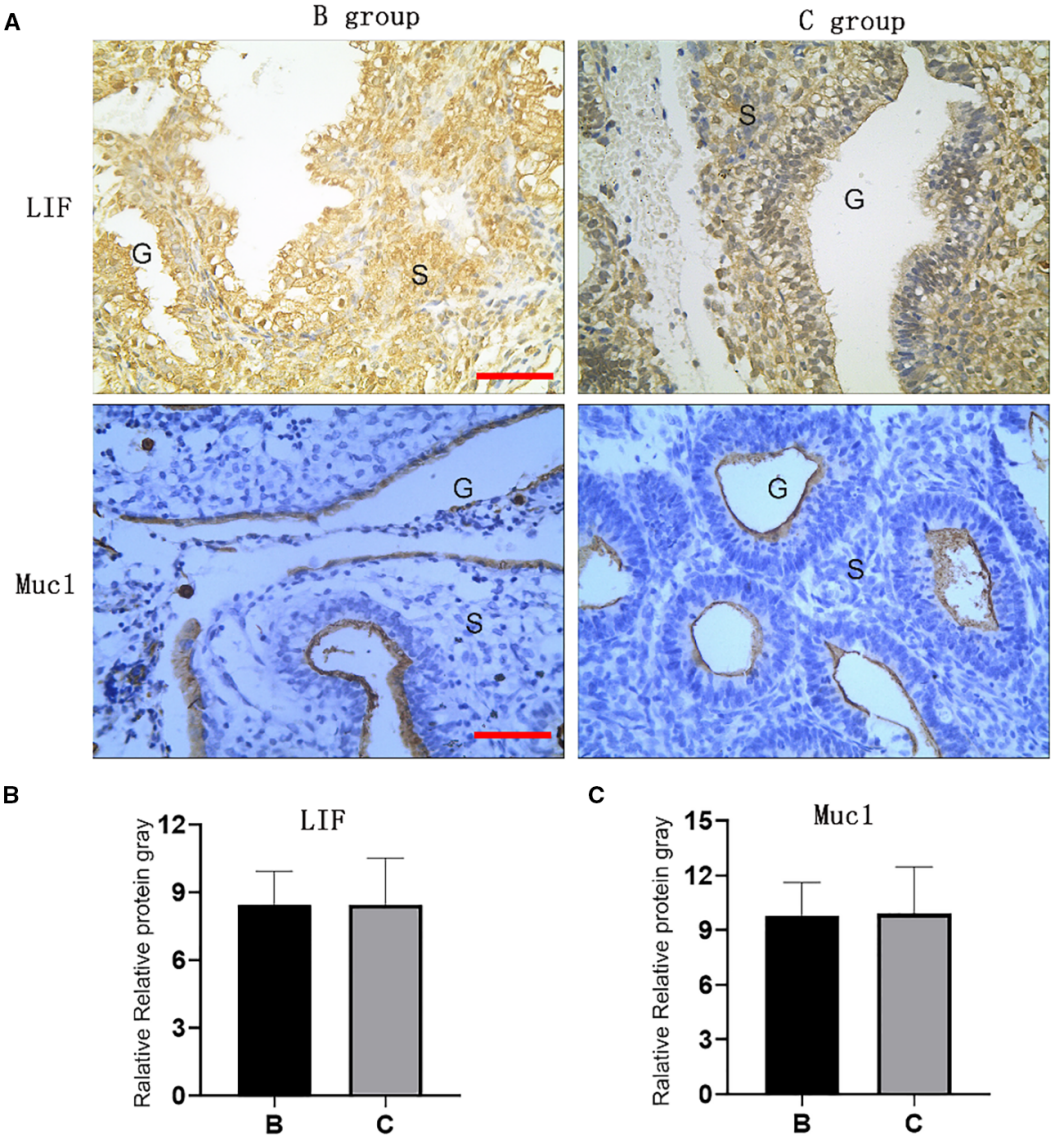
Expression of LIF and Muc1 proteins in implant window endometrium. Expression of LIF and Muc1 proteins in implant window endometrium. **(A)** Immunohistochemical of LIF and Muc1 in endometrium; **(B, C)** Gray values of LIF and Muc1 endometrium immunohistochemical. **(B)** group: oral Micronized 17-beta estradiol hemihydrate; C group: oral and vaginal; G: endometrial glands, S; endometrial stroma; Red line: 1: 100).

The mRNA and protein expression levels of endometrial receptivity markers LIF and Muc1 were comparable between Groups B and C in implant window endometrium, with no statistical differences observed, as shown in **Figures 3**, **4**.

## 4 Discussion

The endometrium serves as the primary target organ of estrogen. During the follicular phase, as follicles develop, ovarian estrogen secretion increases, leading to gradual endometrial thickening—evidence that endometrial proliferation is estrogen-dependent and positively correlated with estrogen levels. Femoston contains Micronized 17-beta estradiol hemihydrate, structurally analogous to natural estradiol, and can be administered orally or vaginally. Vaginal administration effectively elevates local uterine E_2_ concentration, mitigates side effects of oral administration, and offers promise to patients with thin endometria. The study results indicate that combined oral-vaginal administration of Micronized 17-beta estradiol hemihydrate is more conducive to endometrial growth than oral administration alone.

The study findings reveal that oral administration of equivalent doses of Estradiol Valerate Tablets and Micronized 17-beta estradiol hemihydrate yields similar serum E_2_ levels. Notably, when the total dosage of Micronized 17-beta estradiol hemihydrate is maintained, combined oral-vaginal administration increases serum E_2_ levels by approximately 6-fold and endometrial tissue E_2_ levels by ~30-fold compared to oral administration alone. These results align with Tourgeman’s report (8), where vaginal administration of the same Micronized 17-beta estradiol hemihydrate dose resulted in serum Micronized 17-beta estradiol hemihydrate levels ~10-fold higher and endometrial E_2_ concentrations ~70-fold higher than oral administration.

Notably, the marked elevation in estrogen levels raised concerns about its impact on endometrial receptivity, as endometrial receptivity cannot be fully evaluated by endometrial thickness and morphology alone. LIF and Muc1 are established molecular markers of endometrial receptivity (9, 10), and our experimental results indicate that high estrogen levels do not affect the expression of these endometrial receptivity molecules.

Previous studies have indicated that superphysiological estrogen levels may impair endometrial receptivity and IVF clinical outcomes (11), whereas other research has shown that high serum estradiol levels on the hCG administration day do not affect embryo implantation, clinical pregnancy, or spontaneous abortion rates (12). This discrepancy highlights the challenge of defining “excessive” estrogen thresholds. Elevated E_2_ levels are thought to compromise endometrial receptivity primarily by inducing premature serum progesterone elevation, which advances the window of endometrial receptivity (13). However, the impact of serum estradiol (E2) levels on clinical outcomes during hormone replacement therapy (HRT) remains controversial. Shuai J et al. (14) and Wei C et al. (15) demonstrated that elevated serum E2 levels before progesterone administration in HRT-FET cycles are associated with reduced clinical pregnancy rates (CPR) and live birth rates (LBR) after embryo transfer. Conversely, other studies have indicated no correlation between pregnancy outcomes and serum E2 levels on the day of progesterone conversion in HRT-FET cycles (16–19). Specifically, Kong N et al. (16) found that there was no association between clinical pregnancy rate and higher serum E2 levels (exceeding 1400 pg/mL) in HRT-FET cycles. In another study, Deng L et al. (17) further observed that a high E2 level in FET cycles (2005.9 ± 980.0 pg/mL)-achieved through combined oral and vaginal administration of Micronized 17-beta estradiol hemihydrate-does not affect embryo implantation, unlike the impact of high E2 levels in fresh transfer cycles. Moreover, the serum Micronized 17-beta estradiol hemihydrate levels achieved via vaginal administration (2 mg) in this study (1000–2000 pg/ml) were typically lower than those observed during IVF ovulation induction. Additionally, the absence of follicular development in HRT cycles eliminates the risk of premature progesterone surge, explaining why local endometrial E_2_ elevation did not disrupt the expression of receptivity markers like LIF and Muc1.

While vaginal administration of Micronized 17-beta estradiol hemihydrate achieves high E_2_ levels, concerns remain regarding systemic E_2_ bioavailability and its impact on lipid metabolism. Notably, studies have demonstrated that vaginal administration not only enhances E_2_ bioavailability but also elicits no significant changes in SHBG levels or lipid profiles, thereby mitigating potential metabolic adverse effects (20). These findings provide robust support for the clinical utility of vaginal estrogen administration in endometrial preparation for FET cycles.

Notably, the long-term safety of vaginal Micronized 17-beta estradiol hemihydrate and its impact on other metabolic parameters require further investigation. While vaginal administration may enhance endometrial efficacy via local high-concentration E_2_, it potentially increases the risk of endometrial hyperplasia or other estrogen-associated complications. Therefore, before widespread adoption in menopausal hormone replacement therapy, rigorous clinical studies are needed to define its optimal indications and safety profile.

Vaginal administration of Micronized 17-beta estradiol hemihydrate represents a valuable therapeutic strategy for women with refractory thin endometrium unresponsive to other methods. This approach not only effectively enhances endometrial thickness but may also improves pregnancy success rates, offering a novel treatment option for assisted reproductive technology. However, in this study, it is only in the thin endometrium, and whether the vaginal administration of Micronized 17-beta estradiol hemihydrate benefits in the normal thickness of endometrium still needs further study. Notably, vaginal Micronized 17-beta estradiol hemihydrate is not recommended for endometrial preparation in HRT cycles with normal endometrial thickness.

## Data Availability

The original contributions presented in the study are included in the article/supplementary material. Further inquiries can be directed to the corresponding authors.
